# Efficient biosynthesis of ethyl (*R*)-4-chloro-3-hydroxybutyrate using a stereoselective carbonyl reductase from *Burkholderia gladioli*

**DOI:** 10.1186/s12896-016-0301-x

**Published:** 2016-10-18

**Authors:** Xiang Chen, Zhi-Qiang Liu, Chao-Ping Lin, Yu-Guo Zheng

**Affiliations:** 1 Key Laboratory of Bioorganic Synthesis of Zhejiang Province, College of Biotechnology and Bioengineering, Zhejiang University of Technology, Hangzhou, 310014 China; 2 Engineering Research Center of Bioconversion and Biopurification of the Ministry of Education, Zhejiang University of Technology, Hangzhou, 310014 China

**Keywords:** *Burkholderia gladioli*, Carbonyl reductases, Ethyl 4-chloro-3-oxobutanoate, Ethyl (*R*)-4-chloro-3-hydroxybutyrate, Co-expression

## Abstract

**Background:**

Ethyl (*R*)-4-chloro-3-hydroxybutyrate ((*R*)-CHBE) is a versatile chiral precursor for many pharmaceuticals. Although several biosynthesis strategies have been documented to convert ethyl 4-chloro-3-oxobutanoate (COBE) to (*R*)-CHBE, the catalytic efficiency and stereoselectivity are still too low to be scaled up for industrial applications. Due to the increasing demand of (*R*)-CHBE, it is essential to explore more robust biocatalyst capable of preparing (*R*)-CHBE efficiently.

**Results:**

A stereoselective carbonyl reductase toolbox was constructed and employed into the asymmetric reduction of COBE to (*R*)-CHBE. A robust enzyme designed as *Bg*ADH3 from *Burkholderia gladioli* CCTCC M 2012379 exhibited excellent activity and enantioselectivity, and was further characterized and investigated in the asymmetric synthesis of (*R*)-CHBE. An economical and satisfactory enzyme-coupled cofactor recycling system was created using recombinant *Escherichia coli* cells co-expressing *Bg*ADH3 and glucose dehydrogenase genes to regenerate NADPH in situ. In an aqueous/octanol biphasic system, as much as 1200 mmol COBE was completely converted by using substrate fed-batch strategy to afford (*R*)-CHBE with 99.9 % *ee* at a space-time yield per gram of biomass of 4.47 mmol∙L^−1^∙h^−1^∙g DCW^−1^.

**Conclusions:**

These data demonstrate the promising of *Bg*ADH3 in practical synthesis of (*R*)-CHBE as a valuable chiral synthon. This study allows for the further application of *Bg*ADH3 in the biosynthesis of chiral alcohols, and establishes a preparative scale process for producing (*R*)-CHBE with excellent enantiopurity.

**Electronic supplementary material:**

The online version of this article (doi:10.1186/s12896-016-0301-x) contains supplementary material, which is available to authorized users.

## Background

Stereoselective carbonyl reductases (E.C. 1.1.1.x; SCRs) are nicotinamide cofactor-dependent enzymes capable of catalyzing the reversible redox reaction between alcohols and aldehydes/ketones. During the past decade, SCRs have been considerably applied to the synthesis of chiral pharmaceutical intermediates, including anticholesterol drugs [1, 2], *β*-lactams antibiotics [3], anticancer drugs [4], and other important drugs [5]. However, the scale-up of SCR-catalyzed reactions were restricted due to the limited commercially available SCRs, narrow substrate specificity, expensive cofactor dependency, and substrate insolubility.

Ethyl (*R*)-4-chloro-3-hydroxybutyrate ((*R*)-CHBE) is a versatile precursor for pharmacologically valuable products, such as L-carnitine [6], (*R*)-4-amino-3-hydroxybutyric acid (GABOB) [7], and (*R*)-4-hydroxy-pyrrolidone [8]. Several synthetic strategies for optically active CHBE were developed, wherein the enzymatic asymmetric synthesis is the most promising way. Although various biocatalysts have been found to give (*S*)-CHBE [1, 2, 9–13], (*R*)-isomer is in great demand yet less attainable. Since then, several microorganisms and enzymes capable of converting ethyl 4-chloro-3-oxobutanoate (COBE) to (*R*)-CHBE have been documented, including gox2036 from *Gluconobacter oxydans* [14], AKRs from *Sporobolomyces salmonicolor* and *Lodderomyces elongisporus* [15–17], and a reductase from *Bacillus* sp. ECU0013 [18]. However, all of them suffer from impediment such as low substrate concentration, unsatisfactory stereoselectivity, or high substrate/catalyst (S/C) ratio. These shortcomings hindered their applications in the industrial synthesis of (*R*)-CHBE. Exploring more robust SCRs with the ability to prepare enantioenriched (*R*)-CHBE efficiently is thus of great interest.

Herein, we designed and implemented two strategies for identifying novel SCRs, and constructed an enzyme toolbox to screen a robust SCR that can biotransform COBE to (*R*)-CHBE. As the promising SCR, *Bg*ADH3 from *Burkholderia gladioli* CCTCC M 2012379 was selected for further study. The substrate spectrum of *Bg*ADH3 was evaluated toward varied aryl ketones and ketoesters. Furthermore, the practical applicability of *Bg*ADH3 was investigated in the asymmetric synthesis of (*R*)-CHBE using *Escherichia coli* cells co-expressing *Bg*ADH3 and a glucose dehydrogenase (GDH). Since the substrate was poorly soluble and unstable in aqueous environments, biphasic system was established using substrate fed-batch strategy to solve this issue. To our knowledge, this is the first report of SCR from *B. gladioli* subjected to the asymmetric synthesis of enantioenriched (*R*)-CHBE in aqueous/octanol biphasic system.

## Results

### Identification and screening of SCRs

**Fig. 1:**
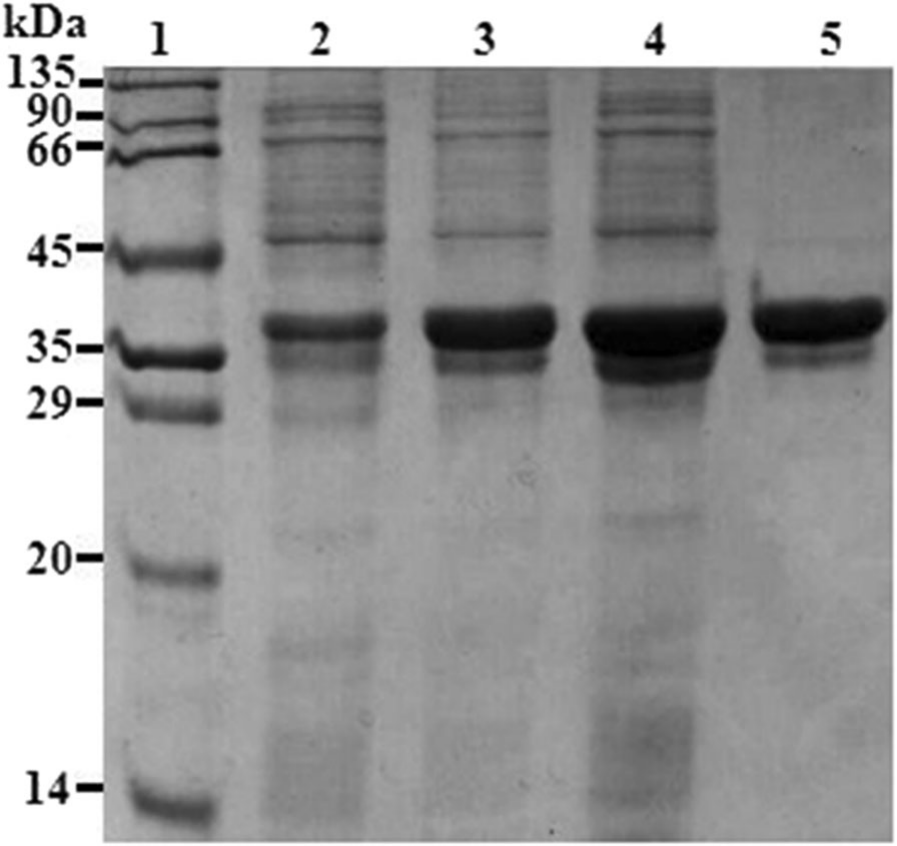
SDS-PAGE analysis of *Bg*ADH3. Lane 1, molecular mass standard; Lane 2, *E. coli* BL21 (DE3)/pET28b-*Bg*ADH3 without IPTG induction; Lane 3, crude extract; Lane 4, soluble fraction; Lane 5, purified *Bg*ADH3

Strain *B. gladioli* CCTCC M 2012379 isolated from soil samples exhibited activity for catalyzing COBE to (*R*)-CHBE (Additional file 1: Table S1). Genome hunting and data mining strategies were selected to discovering robust SCRs from CCTCC M 2012379. Based on bioinformatics analysis of sequence-similarity with gox2036 [14], which is a known NADH-dependent SCR giving enantiopure (*R*)-CHBE. 35 candidates were cloned or synthesized, and heterologously overexpressed in *E. coli* BL21 (DE3), wherein *Bg*ADH3 displayed high activity toward COBE and afforded (*R*)-CHBE (Additional file 2: Table S2). Sequence analysis indicated that the *Bg*ADH3 gene contained an open reading frame with 1011 bp encoding a 336 amino-acid protein, in which the conserved NADP-binding motiff T_97_G_98_XXXG_102_XG_104_ and key catalytic residues N_202_S_228_Y_241_K_245_ were found (Additional file 3: Figure S1). The recombinant *Bg*ADH3 with His_6_-tag mainly presented in the soluble fraction was purified through nickel chelating affinity chromatography [19]. As shown in Fig. 1, the *Bg*ADH3 was not homogeneous, and the estimated molecular mass was around 37 kDa, in accordance with its theoretical value (37.3 kDa). The molecular mass of native *Bg*ADH3 determined on the Discovery BIO GFC 150 column suggested that the quaternary structure of *Bg*ADH3 was dimer.

### Biochemical characterization of *Bg*ADH3

**Fig. 2:**
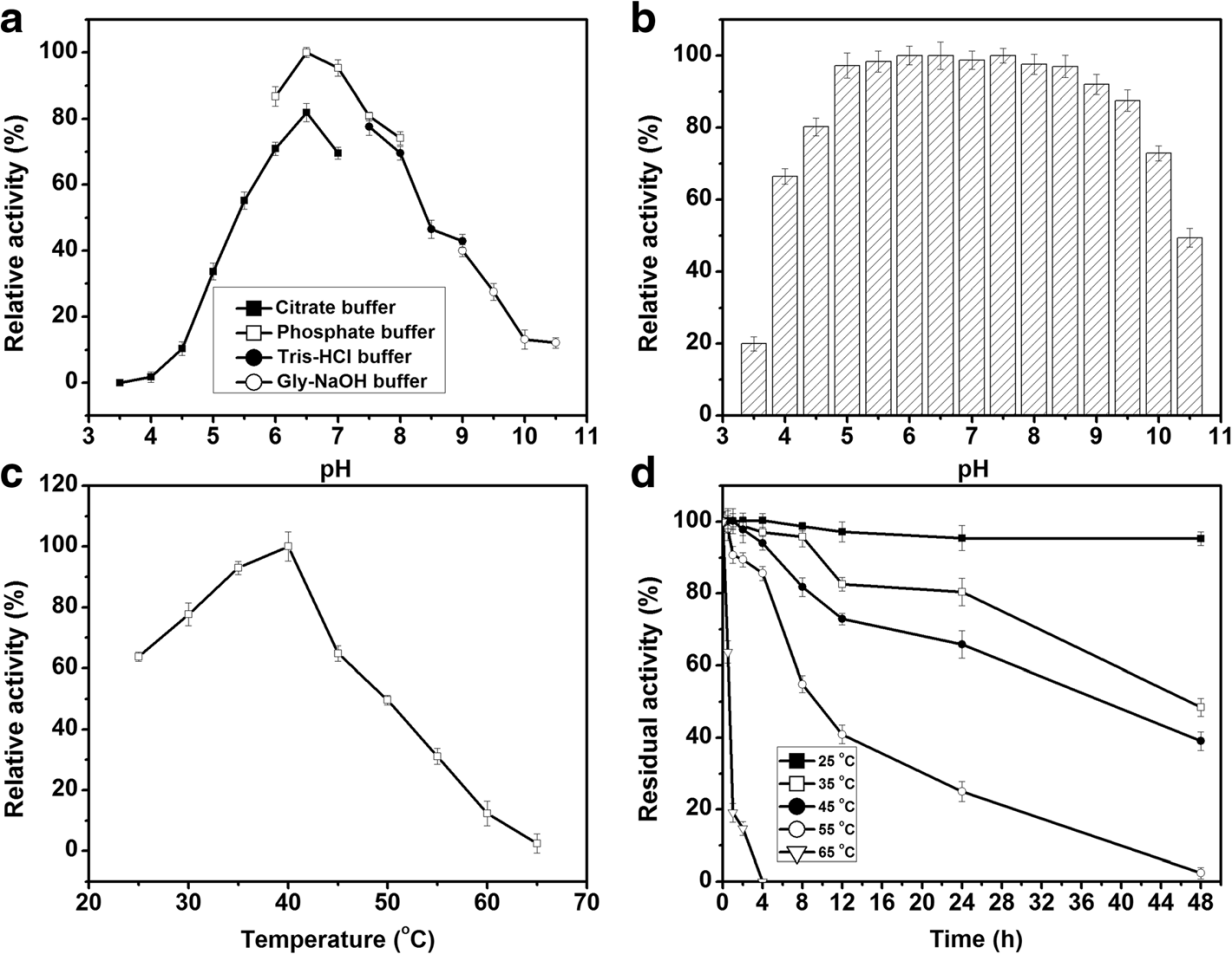
Effects of pH and temperature on the activity and stability of *Bg*ADH3. **a** Optimal pH. **b** pH stability. **c** Optimal temperature. **d** Thermostability. Reaction conditions: COBE (50 mM), NADPH (0.5 mM), and purified *Bg*ADH3 (0.1 mg mL^−1^). The activities were determined after the addition of NADPH for 2 min. All reactions were performed in triplicate

To investigate the effect of pH on the activity and stability of *Bg*ADH3, different pH values ranging from 3.5 to 10.5 were tested. As a result, a bell-shaped pH profile was observed and the maximum activity was found at pH 6.5 (Fig. 2a). Remarkably, the recombinant *Bg*ADH3 is highly stable between pH 5.0 and 9.0 (Fig. 2b). The effect of temperature on *Bg*ADH3 activity was assessed by measuring activity at 25–65 °C. The optimum temperature occurred at 40 °C (Fig. 2c). Thermostability of *Bg*ADH3 was evaluated at temperatures of 25, 35, 45, 55, and 65 °C, respectively. Half-life time of 47 h was found at 45 °C. However, the enzyme was labile at higher temperatures (55 and 65 °C) (Fig. 2d). The presence of Ca^2+^, Co^2+^, Cu^2+^, Fe^3+^, Zn^2+^, and Mg^2+^ slightly enhanced the activity of *Bg*ADH3, whereas Ag^+^ inhibited the activity of the enzyme with a loss of 75 % (Additional file 4: Table S3). When the metal-chelating agent EDTA-Na_2_ was added, the residual activity of *Bg*ADH3 was maintained intact (99 %). Cofactor preference of *Bg*ADH3 was evaluated using COBE as substrate and the result was given in Table 1. The purified *Bg*ADH3 displayed specific activities for the asymmetric reduction of COBE using both NADH and NADPH as cofactors. Although the *K*_mNADPH_ value (0.081 mM) of *Bg*ADH3 was slightly greater than that toward NADH (0.058 mM), the overall catalytic efficiency (*k*_cat_/*K*_m_) for NADPH was approximately 2-fold enhancement compared to that against NADH.

**Table 1:** Apparent kinetic parameters of *Bg*ADH3^a^

Substrate	NADH	NADPH
*K*_m_ (mM)	0.058	0.081
*V*_max_ (μmol min^−1^ mg^−1^)	3.4	9.1
*k*_cat_ (s^−1^)	3.97	10.6
*k*_cat_/*K*_m_ (mM^−1^s^−1^)	68.3	131

^a^Reaction conditions: substrate COBE (50 mM), NADH or NADPH (0.01–0.5 mM), purified *Bg*ADH3 (0.1 mg mL^−1^), pH 6.5, and 40 °C

### Effects of organic solvents

**Fig. 3:**
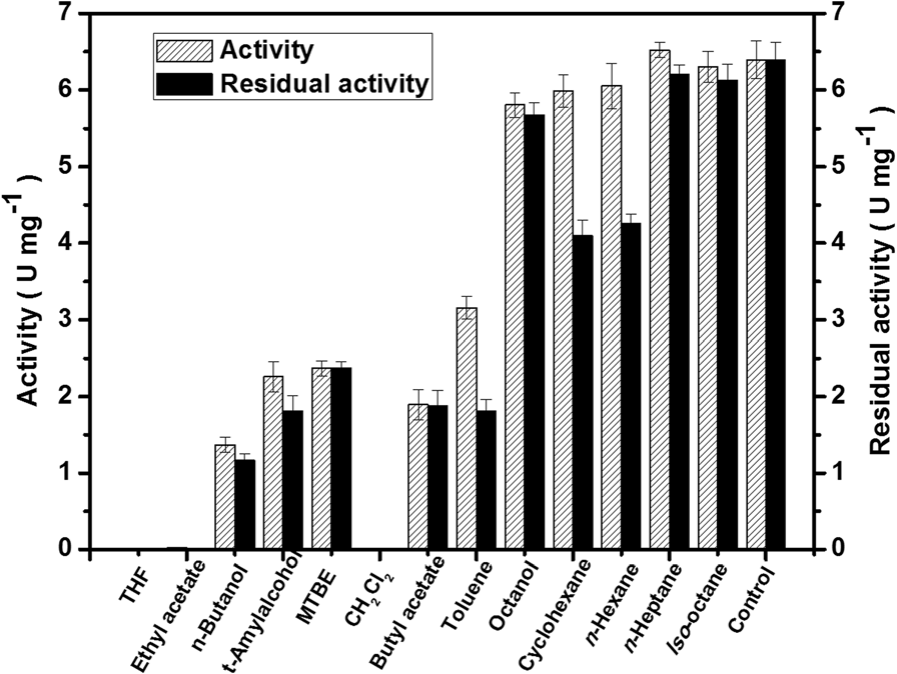
Effect of organic solvents on the activity and stability of recombinant *Bg*ADH3. The activities were measured using COBE as substrate under the standard assay protocol. Stability was determined by measuring the residual activities after incubation with organic solvents at 30 °C for 30 min. The activity in the absence of organic solvent was taken as control. THF, tetrahydrofuran; MTBE, methyl *tert*-butyl ether; CH_2_Cl_2_, dichloromethane

A series of hydrophobic solvents were selected to assess their effects on *Bg*ADH3 activity and histograms representing the residual activities were arrayed in accordance with the increasing log *P* values (Fig. 3). *Bg*ADH3 activity was roughly correlated with the log *P* values of the corresponding solvents, except for dichloromethane, which strongly inhibited the enzyme activity. High activity was observed in the present of hydrophobic solvents with high log *P* value, such as octanol (91 %), cyclohexane (94 %), *n*-hexane (95 %), *n*-heptane (102 %), and *iso*-octane (99 %). Similarly, the effect of the hydrophobicity of the solvents on the retention of *Bg*ADH3 stability was investigated. Satisfactory results were obtained in the present of methyl *tert*-butyl ether (MTBE), butyl acetate, octanol, *iso*-octane, and *n*-heptane, wherein the highest stability was observed in the presence of MTBE (100 %).

### Substrate specificity and stereoselectivity

Specific activity was determined rapidly by using spectrophotometric standard assay described above and the stereoselectivity was evaluated in aqueous phosphate buffer by adding cosubstrate. As shown in Table 2, *Bg*ADH3 were active on all the tested aryl ketones and ketoesters, and exhibited dramatically high activity toward 4’-fluoroacetophenone **6**, 3’,5’-bis(trifluoromethyl) acetophenone **7**, and COBE **12**. In contrast, substrate **4**, **5**, **8**, **15**, **16**, and **17** significantly diminished the activity of *Bg*ADH3. Notably, excellent stereoselectivity of *Bg*ADH3 was also observed for most of the tested substrates, resulting in the corresponding chiral alcohols with >99 % *ee* (Additional file 5: Table S4). *Bg*ADH3 generally exhibited *S* preference, but the corresponding bioproducts with *R* configuration were obtained toward substrates **12**–**15** and **17**.

**Table 2:**
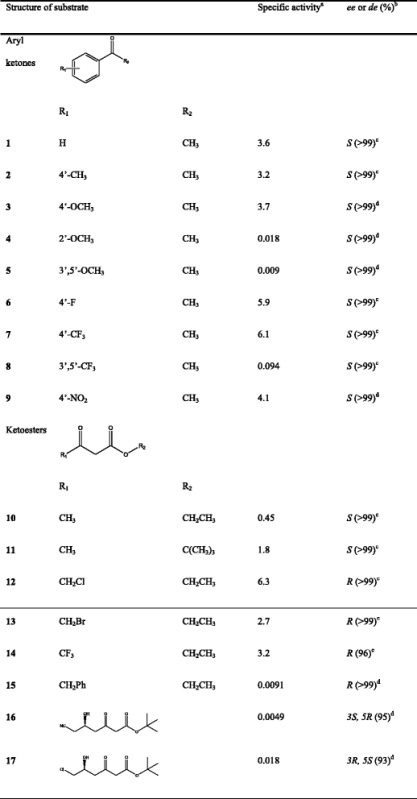
Substrate specificity and stereoselectivity of the *Bg*ADH3

^a^The unit of specific activity was U mg^−1^ protein. ^b^The *ee* or *de* values of the corresponding products. ^c^Determined by chiral GC analysis. ^d^Determined by chiral HPLC analysis

### Co-expression of *Bg*ADH3 and GDH genes

**Fig. 4:**
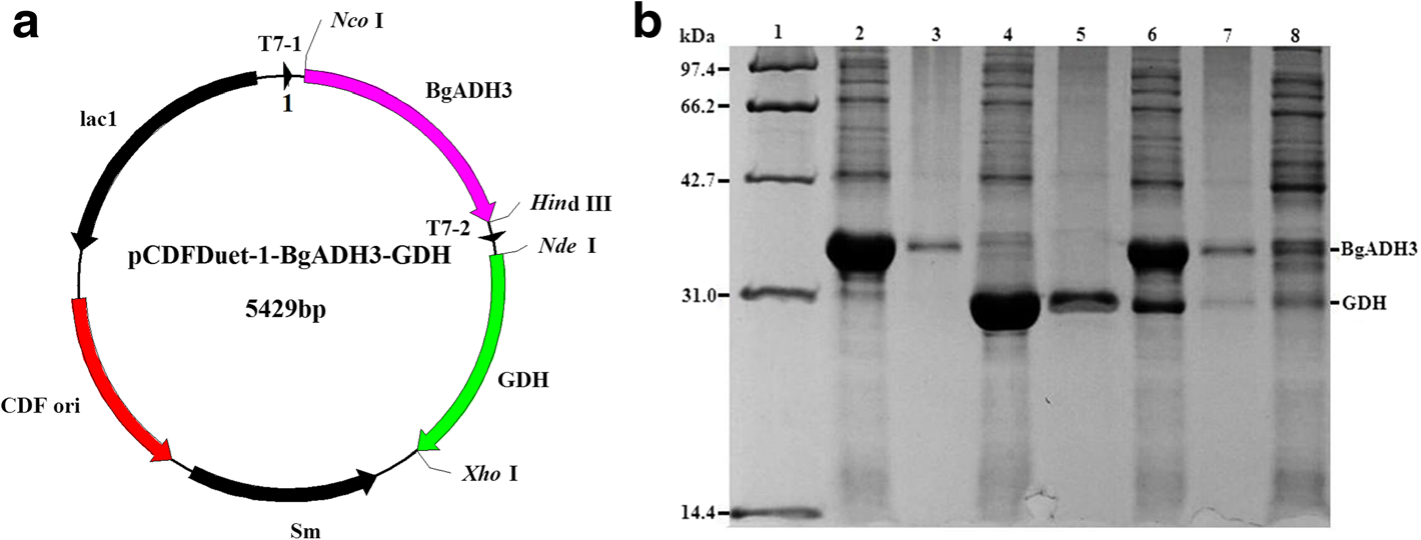
Schematic presentation of the co-expression plasmid contained *Bg*ADH3 and GDH genes and SDS-PAGE analysis. **a** Structure of the co-expression plasmid pCDFDuet-1-*Bg*ADH3-GDH. **b** SDS-PAGE analysis of recombinant co-expressed protein. Lane 1, protein molecular weight marker; Lane 2, soluble fraction of *E. coli*/pET28b-*Bg*ADH3; Lane 3, precipitate of *E. coli*/pET28b-*Bg*ADH3; Lane 4, soluble fraction of *E. coli*/pET28b-GDH; Lane 5, precipitate of *E. coli*/pET28b-GDH; Lane 6, soluble fraction of *E. coli*/pCDFDuet-1-*Bg*ADH3-GDH; Lane 7, precipitate of *E. coli*/pCDFDuet-1-*Bg*ADH3-GDH; Lane 8, *E. coli*/pCDFDuet-1-*Bg*ADH3-GDH without induction

The *Bg*ADH3 gene and GDH gene (786 bp) encoding 261-amino acids were introduced into the MCSI and MCSII sites of pCDFDuet-1 vector (Fig. 4a). After induced by IPTG, the recombinant *Bg*ADH3 and GDH protein were successfully expressed in the same *E. coli* cell. The molecular weights of 37 kDa for *Bg*ADH3 and 28 kDa for GDH was observed in the sodium dodecyl sulfate polyacrylamide gel electrophoresis (SDS-PAGE) (Fig. 4b), which indicated that the expressed products are consistent with the predicted proteins.

### Asymmetric synthesis of (*R*)-CHBE in aqueous-organic solvent system

#### Screening of organic phase

To screen an appropriate organic phase in the biphasic system for (*R*)-CHBE production by using co-expression strain *E. coli*/pCDFDuet-1-*Bg*ADH3-GDH, *n*-butanol, *tert*-amylalcohol, MTBE, butyl acetate, toluene, and octanol were selected to evaluate the effects on product yield and *ee*. The result was presented in Table 3. All yields of aqueous-organic solvents systems were below 50 % or less, except for octanol, in which a yield of 86.6 % was obtained. It was noteworthy that reduction of COBE was strongly inhibited in butyl acetate, affording too low yield (12 %). Interestingly, the enantioselectivity was not impaired and maintained >99 % *ee* in the presence of all the tested organic solvents.

**Table 3:** Screening of organic solvents for biosynthesis of (*R*)-CHBE

Organic solvent	Log *P* ^a^	Partition coefficient^b^		Yield (%)	*ee* (%)
		COBE	CHBE		
*n*-Butanol	0.839	2.6^c^	1.8^c^	37.7	>99
*tert*-Amylalcohol	1.094	3.3^c^	2.9^c^	39.4	>99
MTBE	1.296	8.9^c^	4.8^c^	48.1	>99
Butyl acetate	1.804	10.6	15.8	12.8	>99
Toluene	2.27	21.3	2.8	44.1	>99
Octanol	2.876	5.3	3.8	86.6	>99

^a^Calculated using Advanced Chemistry Development (ACD/Labs) Software V11.02

^b^Partition coefficient is the molar ratio of each compound found in the organic phase to the same compound in the aqueous phase (data from ref. [18, 46, 47])

^c^COBE (73.9 μmol) and CHBE (71.2 μmol) in 0.7 mL phosphate buffer (100 mM, 6.5) were shaken with organic solvents (0.7 mL) at 30 °C in an eppendorf tube. The concentrations of COBE and CHBE in each phase were determined by GC analysis

#### Optimization of reaction conditions

**Fig. 5:**
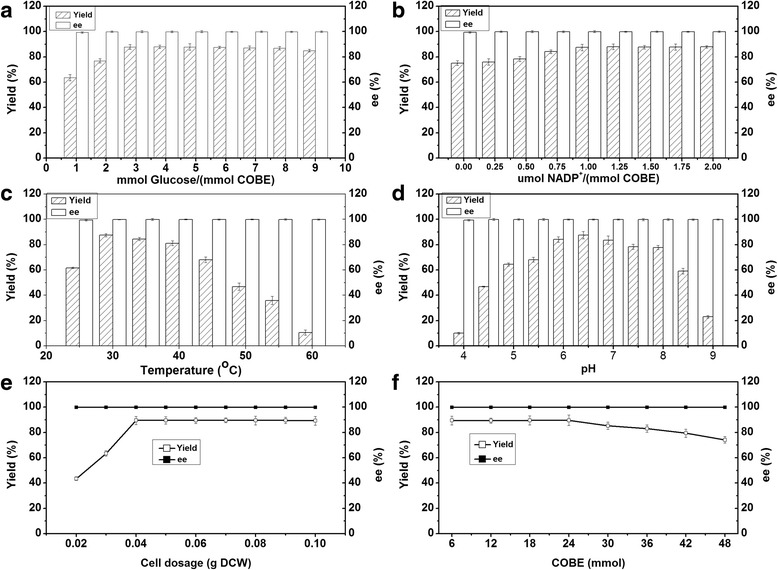
Effects of glucose concentrations (**a**), NADP^+^ concentrations (**b**), temperature (**c**), pH (**d**), cell dosage (**e**), and substrate loading (**f**) on the biosynthesis of (*R*)-CHBE using co-expression *E. coli* cells

To improve the asymmetric reduction of COBE to (*R*)-CHBE by using co-expression *E. coli*, different reaction conditions were further investigated. As shown in Fig. 5a, it was found that the yield of (*R*)-CHBE was significantly improved when the glucose/COBE (mmol/mmol) ratio was increased to 3, and then was clearly diminished when the ratio was over 8. To effectively synthesize (*R*)-CHBE, the amount of NADP^+^ was also optimized. The highest yield was obtained using a NADP^+^/COBE (μmol/mmol) ratio of 1.0 and a huge excess of NADP^+^ was not necessary (Fig. 5b). The maximum level of yield was found at 30 °C and pH 6.5 (Fig. 5c and d). An increased production of (*R*)-CHBE was observed as the cell dosage was raised from 0.02 to 0.04 g DCW. When the cell dosage was excess 0.04 g DCW, the yield of (*R*)-CHBE was not increased significantly (Fig. 5e). Keeping constant the S/C (mmol/g DCW) ratio of 150, different amount of COBE were further estimated. The yield of (*R*)-CHBE was significantly decreased when more than 24 mmol of the substrate was added (Fig. 5f). Based on the above results, the optimum reaction conditions in 20 mL system were obtained: COBE 24 mmol, glucose 72 mmol, NADP^+^ 24 μmol, cell dosage 0.16 g DCW, pH 6.5, and 30 °C.

#### Substrate fed-batch strategy and preparative scale

**Fig. 6:**
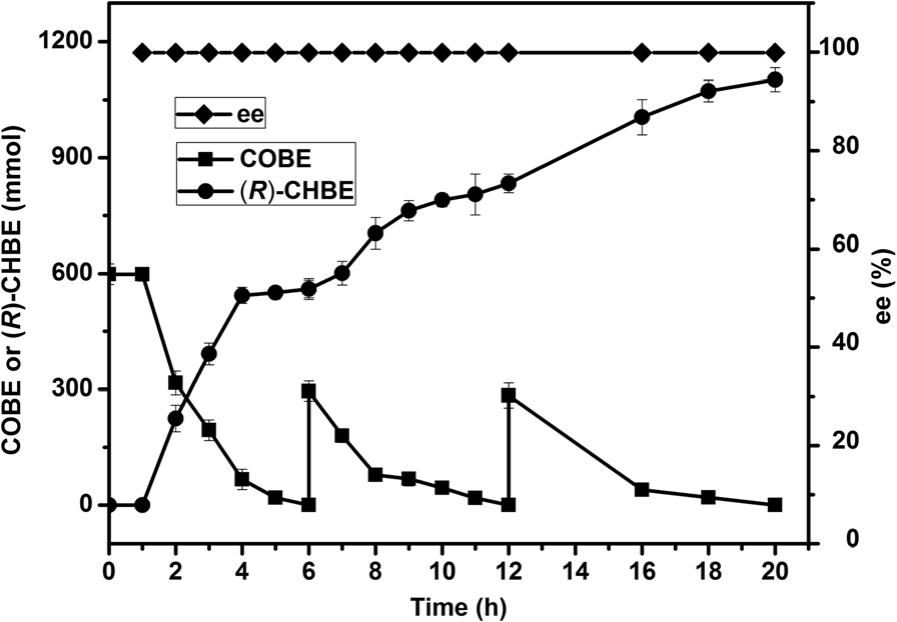
Production of (*R*)-CHEB using co-expression *E. coli* cells in a water/octanol biphasic system with a substrate fed-strategy. Reduction of COBE using sonicated co-expressed *E. coli* cells (8 g DCW) was conducted at 30 °C in 0.5 L phosphate buffer (pH 6.5) containing glucose (1800 mmol) and COBE (600 mmol) and 0.5 L octanol. COBE (300 mmol) together with 3 equiv. glucose were added to the system at 6 h and 12 h, respectively

The reaction was performed in a water/octanol biphasic system with a total of 1200 mmol COBE addition that was fed in three steps, during which the resulting gluconic acid was neutralized with 2 M NaOH to maintain an initial pH of 6.5. As shown in Fig. 6, at 20 h, 1101.6 mmol of (*R*)-CHBE was produced with a satisfactory enantiomeric purity of 99.9 % *ee* and the remaining COBE substrate was not detectable. After normal downstream processing, the desired chiral alcohol product was obtained with a yield of 91.8 %. To further confirm the structure and configuration of the final product, NMR, MS, and optical rotation were performed. ^1^H NMR (400 MHz, CDCl_3_): *δ* 4.3–4.28 (m, 1H), 4.18 (q, *J* = 7.2 Hz, 2H), 3.60–3.61 (m, 2H), 3.01 (s, 1H), 2.57–2.66 (m, 2H), and 1.24–1.29 (m, 3H) (Additional file 6: Figure S2); ^13^C NMR (126 MHz, CDCl_3_): *δ* 171.76, 67.93, 60.98, 48.12, 38.51, 14.07 (Additional file 6: Figure S3); MS calculated for C_6_H_12_ClO_3_: 167.0, found: 167.2 [M + 1]; [α]^25^_D_ = +15.1° (c = 20 mg/mL, CHCl_3_) ([α]^23^_D_ = + 14° (neat), Sigma-Aldrich).

## Discussion

During the past decade, the efficiency of asymmetrically biocatalytic synthesis catalyzed by SCRs has emerged as an environmentally sustainably alternative to traditional organo- and metallocatalysis due to the inherent high stereoselectivity of the biocatalysts and the mild reaction conditions. However, there are still impeded in their large-scale application due to the limited commercially robust biocatalysts, the narrow substrate specificity and the dependency of expensive cofactor as well as the water-insoluble substrates [20]. With the development of genomics, proteomics, and bioinformatics, genome hunting and data mining have become powerful tools for exploiting novel enzymes [21, 22]. In the present study, on the basis of screening results of microorganisms, and the combination of the BLASTp searching using the reported carbonyl reductase (GenBank accession no. AAW61772.1), a toolbox contained 35 candidates predicted as carbonyl reductases was successfully constructed, and further subjected to the asymmetric synthesis of enantioenriched (*R*)-CHBE.

Optically active (*R*)-CHBE is a useful chiral building block for L-carnitine, a biomolecule capable of modulating the serum oxidation stress and lipid profile [23], and is synthesized through the asymmetrical reduction of COBE. A SCR from *G. oxydans* 621H was reported to provide for accessing (*R*)-CHBE, but with low substrate concentration (6 mM) [14]. Using an AKR from *S. salmonicolor*, as much as 268 g L^−1^ of COBE was converted to (*R*)-CHBE yet with moderate enantioselectivity (only 91.7 % *ee*) [15]. A reductase from *Bacillus* sp. ECU0013 was capable of producing (*R*)-CHBE with high substrate loading (215 g L^−1^) and enantioselectivity (99.6 % *ee*), however, it required high S/C and only small-scale was accomplished [18]. In this study, a robust SCR designed as *Bg*ADH3 (GenBank Accession No. AEA63541) from *B. gladioli* CCTCC M 2012379 demonstrated high catalytic efficiency and excellent enantioselectivity thus was considered as a promising SCR for the asymmetric synthesis of optically active (*R*)-CHBE, accomplishing an economic and environmental balance.

Considering the majority of pairwise similarities between SCR members are in the range of 15–30 %, and the presence of conserved NADP-binding motiff TGXXXGXG as well as N-S-Y-K catalytic terads [24], *Bg*ADH3 was defined as the ‘classical’ short-chain dehydrogenases/reductases (SDR). Although the well-known *Lactobacillus brevis* SDR exhibited strong Mg^2+^ dependency [25], SDRs generally does not require metal ions. In this case, the metal chelator EDTA did not impact the activity, and most of the tested metal ions did not significantly improved *Bg*ADH3 activity, revealing that *Bg*ADH3 is typical of non-metal SDR. Several SCRs have been described capable of oxidizing both NADH and NADPH in the asymmetric reduction [26, 27]. In this study, cofactor preference of *Bg*ADH3 was evaluated using COBE as substrate, indicating that *Bg*ADH3 was not completely specific to NADH or NADPH as well. Due to the higher catalytic efficiency, NADPH was selected as the cofactor in the reduction of COBE using *Bg*ADH3. Notably, the catalytic ability of *Bg*ADH3 was approximately 5-fold improvement compared to that found on the literature for gox2036, an NADH-dependent SDR provided access to (*R*)-CHBE [14].

Enzymes generally require aqueous media in which the hydrophobic substrates are poorly soluble and even unstable. A straightforward solution to this issue might be the application of an aqueous organic solvent system [28, 29]. There are several documented cases in which organic solvents can affect the enzymatic activity [30–33]. Compared to hydrophobic solvents, hydrophilic ones are usually easier impact the activity due to the greater tendency of stripping tightly bound water in the enzyme molecules [34]. Herein, batteries of water-immiscible solvents were tested for the influence on the activity of *Bg*ADH3. High activity observed in the presence of hydrophobic solvents with high log *P* value was consistent with the recent study of organic tolerance of alcohol dehydrogenases [35]. In contrast, the stability of *Bg*ADH3 showed no dependence on the hydrophobicity of solvents. This finding is in agreement with the assumption that solvent functionality is also significance for enzyme deactivation [36]. Thus, it was established that log *P* was not an adequate parameter to describe the trend of enzyme tolerance to organic solvents. Although *Bg*ADH3 exhibited high activity and stability in the presence of cyclohexane, *n*-hexane, *n*-heptane, and *iso*-octane, COBE and (*R*)-CHBE exhibited low solubility due to the poor partition coefficient thus were not suitable for the application in aqueous organic solvent system. Butyl acetate has been frequently introduced as the non-aqueous phase in the reduction of COBE [2, 9], however, *Bg*ADH3 was strongly inactivated in this solvent. In this case, octanol was eventually chosen as the organic phase in the biphasic system.

Different position and size of the substituents in carbonyl compounds play an critical role in the enzyme activity [37]. With regard to acetophenone derivatives, the *ortho*-substituted group (**4**) or multi-substitution (**5** and **8**) reduced the *Bg*ADH3 activity due to the steric hindrance effects on the hydrogen attack from NADPH to the carbonyl group. Likewise, the bulky substituents in ketoesters adjacent to the carbonyl group also have negative effects. *Bg*ADH3 generally obeys Prelog’s rule with *S* preference by the hydride transfer from the cofactor to the Re-face of the carbonyl group during the enzymatic reductions, similar to the majority of known SCRs [14]. The inverted stereochemical assignment observed toward **12**–**14** and **17** was explained by a higher Cahn-Ingold-Prelog priority in the smaller side of substrate than the large one, thus still followed the Prelog’s rule of hydride delivery. However, the reduction of **15** catalyzed by *Bg*ADH3 also afforded the (*R*)-enantiomer, suggesting that bulky substituent might affect the configuration of the corresponding alcohol.

Although the upscale application of SCRs was impeded by the requirement for expensive cofactors such as NAD(P)^+^/NAD(P)H, enzyme-coupled and substrate-coupled cofactor recycling strategies have been developed for surmounting this challenge [38–40]. Compared to the substrate-coupled cofactor recycling with reversibility and poor thermodynamic driving force, enzyme-coupled cofactor regeneration exhibited more efficient and inexpensive. In this study, GDH from *Exiguobacterium sibiricum* 255*–*15 (GenBank: ACB59697.1) and glucose was successfully coupled with *Bg*ADH3 in the biosynthesis of (*R*)-CHBE. Expressing SCR and GDH in same cell is the possibility to avoid using an additional expensive cofactor when performing the biotransformation with high substrate loading [41–43]. Herein, the *Bg*ADH3 and GDH genes were introduced into the pCDFDuet-1 vector to construct the co-expressed plasmid pCDFDuet-1-*Bg*ADH3-GDH and strain *E. coli*/pCDFDuet-1-*Bg*ADH3-GDH. The yield was enhanced approximately 17 % in the presence of 1.0 μmol NADP^+^/(mmol COBE) or more compared to that in the absence of external NADP^+^, likely because the amount of cofactor in vivo is insufficient to this reaction. Based on cost considerations, the ratio of NADP^+^/COBE (μmol/mmol) was limited to 1.0. Reaction pH and temperature play important roles in biotransformation, which can significantly affect the activity and stability of biocatalysts, the speed of substrate molecules motion, and the activation energy of the bioreduction. The optimum temperature of co-expressed *E. coli* was inconsistent with that of single *Bg*ADH3. This might be explained that the high temperature impact the GDH activity before affect the *Bg*ADH3 activity. The substrate loading was considerably improved with the increased of cell dosage at a fixed S/C (mmol/g DCW) of 150, while the yield was significantly reduced when the cell dosage was too high due to the increased viscosity in the reaction media which might be hinder the molecular transfer between substrate and enzyme [1]. To the best of our knowledge, this is the first report of a preparative-scale highly stereoselective reduction of COBE to (*R*)-CHBE by using a newly cloned *Bg*ADH3 co-expressed with GDH. From a practical viewpoint, this asymmetric synthesis of (*R*)-CHBE is promising since no example reported to produce (*R*)-CHBE with more than 1200 mmol of in 1-L reaction system yet (Table 4).

**Table 4:** Comparison of several reported biocatalysts for producing (*R*)-CHBE

Enzyme	SsAR	SsALR	CpSADH	BYueD	Gox2036	LEK	*Bg*ADH3
Family	AKR	AKR	MDR	-	SDR	AKR	SDR
Solvent system	water/*n*-butyl acetate	water	water	water/toluene	water	water	water/octanol
Cofactor enzyme	GDH	GDH	/	GDH	GDH	GDH	GDH
Cosubstrate	G	G	IPA	G	G	G	G
External cofactor	NADP^+^	NADP^+^	Without	NADP^+^	NAD(H)	Without	NADP^+^
Substrate loading (mmol)	91	0.61	1.525	13	0.06	111	1200
Biocatalyst (g)	2 (wet)	0.4 (wet)	/	0.5 (dry)	0.1 (wet)	17 (dry)	8 (dry)
Reaction volume (mL)	50	10	25	20	10	/	1000
Reaction time (h)	50	10	17	5	24	1	20
*ee* of (*R*)-CHBE	91.7	99	99	99.6	100	>99	99.9
References	[15]	[48]	[49]	[18]	[14]	[17]	This work

## Conclusions

In summary, a SCR toolbox was constructed in this work through genome hunting and data mining approaches based on bioinformatics analysis of sequence-similarity with known stereoselective SCR, and further subjected to the asymmetric synthesis of (*R*)-CHBE. The newly cloned *Bg*ADH3 from *B. gladioli* displayed high activity and excellent enantioselectivity toward COBE, and served as a versatile SCR with a broad substrate spectrum converting varied aromatic ketones and ketoesters to the corresponding chiral alcohols with excellent enantiomeric purity (>99 % *ee*). Furthermore, asymmetric synthesis of (*R*)-CHBE was developed by using recombinant *E. coli* cells co-expressing *Bg*ADH3 and GDH. Using substrate fed-batch mode and aqueous/octanol biphasic system, the decomposition of COBE and its inhibition of the biocatalyst were significantly alleviated. Using the “designer cells”, (*R*)-CHBE was produced at a high space-time yield per gram of biomass of 4.47 mmol∙L^−1^∙h^−1^∙g DCW^−1^ and excellent enantiopurity (99.9 % *ee*) with the highest substrate total loading of 1200 mmol in 1-L reaction system reported so far. This study allows for the further application of *Bg*ADH3 in the biosynthesis of chiral alcohols, and establishes a preparative scale process for producing (*R*)-CHBE with excellent enantiopurity.

## Methods

### Strains, plasmids and chemicals

The *E. coli* DH5α (Tiangen biotech Co., Ltd., Beijing, China) and *E. coli* BL21 (DE3) (Novagen, Darmstadt, Germany) were selected as hosts in this study. Plasmid pGEM-T (Promega, Beijing, China) and pET28a (Novagen, Darmstadt, Germany) were used for cloning and expression, respectively. Polymerase chain reaction (PCR) mix and restriction endonucleases were purchased from Vazyme (Nanjing, China). Ampicillin and kanamycin were obtained from solarbio (Beijing, China). Streptomycin and IPTG were purchased from Sangon Biotech (Shanghai, China). COBE and (*R*)-CHBE were provided by J&K Scientific Ltd. (Shanghai, China). All other chemicals were of analytical grade and commercially available.

### Expression of SCRs in *E. coli* cells

Strain CCTCC M 2012379 was cultivated as described previously [44]. The genomic DNA of this strain was extracted using a FastDNA® Spin Kit for Soil (MPBio, Shanghai, China). The genes of SCRs were amplified by PCR using appropriate primers designed according to the putative proteins from *B. gladioli* BSR3 (Additional file 7: Table S5). Each amplified DNA fragment was inserted into pGEM-T to create pGEM-T-SCR, and digested with restriction endonucleases. The desired fragment was subcloned into linearied pET28a with the same restriction enzyme cutting sites and transformed in *E. coli* BL21 (DE3). Other SCRs genes were synthesized and transformated in *E. coli* BL21 (DE3).

### Cell growth and enzyme purification

The recombinant *E. coli* strains with SCRs were cultivated, induced, and purified as described previously [45]. The protein expression level was verified by SDS-PAGE. The native molecular weight was measured using a Discovery BIO GFC 150 (300 × 7.8 mm, 3 μm) column (Sigma-Aldrich, Shanghai, China) equilibrated with phosphate buffer (150 mM) at 1.0 mL/min using cytochrome c (12.4 kDa), ovalbumin (43 kDa), bovine serum albumin (66 kDa), and yeast alcohol dehydrogenase (150 kDa) as standard proteins. Protein concentration was determined using BCA Protein Assay Kit (KeyGEN BioTECH, Nanjing, China) with bovine serum albumin (BSA) as a standard.

### Enzyme assays

Specific activity was detected spectrophotometrically by monitoring the depletion in the absorption of NAD(P)H at 340 nm. One unit (U) of enzymatic activity was defined as 1 μmol of NAD(P)H consumed per minute under the assay conditions. Each assay mixture contained COBE (50 mM) and NAD(P)H (0.5 mM) in phosphate buffer (100 mM, pH 6.5). The activity was determined after the addition of NAD(P)H for 2 min. The same method was used in all reactions against other substrates.

### Characterization of recombinant *Bg*ADH3

Specific activities were evaluated under standard assay conditions using COBE as substrate. The effect of pH on the activity of *Bg*ADH3 was assessed within a pH range of 3.5–10.5, using disodium hydrogen phosphate-citrate buffer (3.5–7.0), phosphate buffer (6.0–8.0), Tris–HCl buffer (7.5–9.0), and Gly-NaOH buffer (9.0-10.5). The pH stability was tested by pre-incubating purified *Bg*ADH3 in buffers with different pH values at 30 °C for 2 h, and then the residual activity was measured. The non-incubated enzyme was considered as a control. The effect of temperature on the activity was studied by assaying activities at temperatures ranging from 25 °C to 65 °C. The thermostability was estimated by incubating the purified *Bg*ADH3 in the phosphate buffer (100 mM, pH 6.5) at 25, 35, 45, 55, and 65 °C, respectively. Samples were withdrawn per hour and the residual activities were detected. The non-heated enzyme was taken as a control. The effect of metal ions on the activity was evaluated by pre-incubating recombinant *Bg*ADH3 in the presence of Fe^2+^, Ni^2+^, Fe^3+^, Ca^2+^, Ba^2+^, Cu^2+^, Mn^2+^, Zn^2+^, Co^2+^, Mg^2+^, Ag^+^, and EDTA (2 mM) at 30 °C for 30 min. Measurements of kinetic parameters were done by altering the concentration of NADH or NADPH (0.01–0.5 mM) at a constant COBE concentration (50 mM), and were calculated through nonlinear regression of the Michaelis-Menten equation. The influence of organic solvents on the activity was assessed by adding hydrophobic solvents (50 %, v/v), including tetrahydrofuran (THF), ethyl acetate, *n*-butanol, *tert*-amylalcohol, MTBE, dichloromethane, butyl acetate, toluene, octanol, cyclohexane, *n*-hexane, *n*-heptane, and *iso*-octane. Stability was determined by measuring the residual activity after incubation with organic solvents at 30 °C for 30 min. All assays were performed in triplicate.

### Stereoselectivity

The stereoselectivity of *Bg*ADH3 was estimated by testing the asymmetric reduction of aryl ketones and ketoesters using glucose as the cosubstrate. Each reaction mixture was comprised of phosphate buffer (100 mM, pH 6.5), substrate in DMSO (50 mM, 5 % v/v), NADPH (0.05 mM), glucose (278 mM), GDH (0.1 mg mL^−1^), and purified *Bg*ADH3 (0.1 mg mL^−1^) in a total volume of 0.5 mL, and proceeded at 40 °C for 6 h. Reaction mixture without recombinant *Bg*ADH3 was used as the control. After complete of the reaction, each biotransformation solution was extracted with ethyl acetate. The *ee* or *de* values of products were determined by GC or HPLC analysis.

### Co-expression of *Bg*ADH3 and GDH genes

The pGEM-T-GDH was constructed as previously reported [9], and digested with *Nde* I and *Xho* I restriction enzymes. The fragment of interest was induced into the *Nde* I/*Xho* I site (MCSI) of plasmid pCDFDuet-1 to afford recombinant pCDFDuet-1-GDH vector. Subsequently, the *Bg*ADH3 gene cut from pGEM-T-*Bg*ADH3 with *Nco* I and *Hin*d III was ligated to the *Nco* I/*Hin*d III site (MCSII) of pCDFDuet-1-GDH, leading to the final recombinant plasmid pCDFDuet-1-*Bg*ADH3-GDH harboring *Bg*ADH3 and GDH genes. The resulting co-expressed plasmid was further transformed into *E. coli* BL21 (DE3) and grown in LB medium containing streptomycin (50 μg mL^−1^). The expression levels of this recombinant genes were verified by SDS-PAGE.

### Efficient biosynthesis of (*R*)-CHBE in aqueous-organic solvent system

#### Screening of organic solvents

Different organic solvents were evaluated in the asymmetric synthesis of (*R*)-CHBE using co-expression *E. coli*/pCDFDuet-1-*Bg*ADH3-GDH at 40 °C for 6 h by mixing an equal volume of the tested solvents with 10 mL phosphate buffer (100 mM, pH 6.5) consisted of COBE (6 mmol), glucose (12 mmol), NADP^+^ (6 μmol), and sonicated co-expressed *E. coli* cells (0.04 g DCW). The pH of the reaction mixture was monitored at 6.5 with 2 M NaOH during reaction. The *ee* and quantity of COBE and (*R*)-CHBE were determined by GC [2].

#### Optimization of reaction conditions

Several reaction parameters (e.g., glucose concentration, NADP^+^ concentration, reaction pH, reaction temperature, cell dosage and substrate loading, etc.) affected the biotransformation were assessed by adding different concentrations of glucose (1–9 mmol glucose/(mmol COBE)), NADP^+^ (0–2 μmol/(mmol COBE)), cell dosage (0.02–0.1 g DCW), and COBE (6–48 mmol) in 10 mL phosphate buffer mixed with an equal volume of octanol. Unless otherwise stated, each assay was stirred at 40 °C for 6 h. The effects of reaction temperature and pH on the biosynthesis were performed at various temperatures (25–60 °C) and different pHs (4.0–9.0).

#### Substrate fed-batch strategy and preparative scale

Substrate fed-batch was carried out in 1-L reaction system, which comprising phosphate buffer (100 mM, pH 6.5), COBE (600 mmol), glucose (1800 mmol), and sonicated *E. coli*/pCDFDuet-1-*Bg*ADH3-GDH (8 g DCW), in a final volume of 0.5 L, was mixed with an equal volume of octanol in a bioreactor with two electrodes for monitoring the temperature and pH. The resulting mixture was stirred at 200 rpm for 20 h. The temperature was maintained at 30 °C and the pH was adjusted to 6.5 with 2 M NaOH during the reaction. COBE (300 mmol) and glucose (900 mmol) were added to the reaction mixture at 6 and 12 h, respectively. After reaction, the two layers were separated and the aqueous layer was extracted twice with ethyl acetate. The extracted layers were combined with the original organic layer, washed with NaHCO_3_, and dried by anhydrous Na_2_SO_4_. The obtained solvent was evaporated under vacuum, offering the final product as oily liquid. High-resolution mass spectrum (MS) was obtained on an Agilent 6210 TOF LC/MS system (Palo Alto, CA, USA). Nuclear Magnetic Resonance (NMR) spectra of the final product were performed on Bruker AVANCE III (Bruker, Switzerland) spectrometer. Polarimetry were carried on Rudolph Autopol IV polarimeter (Rudolph, USA) using the sodium D line (589 nm) at 25 °C.

## Additional files

Additional file 1: Table S1. Results of screening microorganisms for direct asymmetric reduction of ethyl 4-chloro-3-oxobutanoate. (DOCX 24 kb)
Additional file 2: Table S2. Results of screening stereoselective carbonyl reductases for direct asymmetric reduction of ethyl 4-chloro-3-oxobutanoate. (DOCX 18 kb)
Additional file 3: Figure S1. Amino acid sequence alignments of short-chain alcohol dehydrogenase/reductase using ESPript 3.0. Sequences are *Burkholderia gladioli* from this study (*Bg*ADH3, GenBank Accession No. AEA63541), *Gluconobacter oxydans* 621H (Gox2036, GenBank Accession No. AAW61772.1), *Lactobacillus brevis* (*Lb*ADH, GeneBank Accession No. CAD66648.1), *Salmonella enteric* (Ygha, PDB: 3R3S_A), and *Bacillus anthracis* (*Ba*ADH, PDB: 3I3O_A). The cofactor-binding motifs in the SDRs, TGXXXGXG and PG, are highlighted in orange and triangle while the residues of the catalytic tetrad (N, S, Y, and K) are highlighted in green. (DOCX 212 kb)
Additional file 4: Table S3. Effect of metal ions on the activity of recombinant *Bg*ADH3. (DOCX 17 kb)
Additional file 5: Table S4. GC and HPLC analysis of the *ee* or *de* values of products. (DOCX 19 kb)
Additional file 6: Figure S2.
^1^H NMR of the isolated (*R*)-CHBE (500 MHz, CDCl_3_). **Figures S3.** ^13^C NMR of the isolated (*R*)-CHBE (126 MHz, CDCl_3_). (DOCX 1122 kb)
Additional file 7: Table S5. Primers from *Burkholderia gladioli* CCTCC M 2012379 for PCR amplification. (DOCX 18 kb)

## Abbreviations

(*R*)-CHBE: Ethyl (*R*)-4-chloro-3-hydroxybutyrate
CH_2_Cl_2_: Dichloromethane
COBE: Ethyl 4-chloro-3-oxobutanoate
*E. coli*: *Escherichia coli*
GDH: Glucose dehydrogenase
MS: Mass spectrum
MTBE: Methyl *tert*-butyl ether
NMR: Nuclear magnetic resonance
SCRs: Stereoselective carbonyl reductases
SDS-PAGE: Sodium dodecyl sulfate polyacrylamide gel electrophoresis
THF: Tetrahydrofuran
