# Supplementary material for: Efficient biosynthesis of ethyl (R)-4-chloro-3-hydroxybutyrate using a stereoselective carbonyl reductase from Burkholderia gladioli
Source: BMC Biotechnol. 2016 Oct 18;16:70. doi: 10.1186/s12896-016-0301-x (PMC5070160; doi:10.1186/s12896-016-0301-x)
Supplement: Additional file 1: Table S1. — Results of screening microorganisms for direct asymmetric reduction of ethyl 4-chloro-3-oxobutanoate. (DOCX 24 kb) [file 12896_2016_301_MOESM1_ESM.docx]

**Additional file 1: Table S1.** Results of screening microorganisms for direct asymmetric reduction of ethyl 4-chloro-3-oxobutanoate.

| Strains^a^ | (*R*)-CHBE | (*S*)-CHBE |
| --- | --- | --- |
| *Burkholderia gladioli* CCTCC M 2012379 | + | + |
| *Candida krusei* CCTCC M 2010335 | - | - |
| *Pseudomonas aeruginosa* CCTCC M 2011394 | - | - |
| *Kluyveromyces lactis* CCTCC M 2014380 | - | + |
| *Candida albicans* CCTCC M 2014382 | - | + |

Notes: +, positive; −, negative. ^a^ ref. [[1-5](#_ENREF_1)].

1. Chen X, Zheng YG, Liu ZQ, Sun LH. Stereoselective determination of 2-benzamidomethyl-3-oxobutanoate and methyl-2-benzoylamide-3-hydroxybutanoate by chiral high-performance liquid chromatography in biotransformation. J Chromatogr B. 2015;974:57-64.

2. Luo X, Wang YJ, Zheng YG. Cloning and characterization of a NADH-dependent aldo-keto reductase from a newly isolated *Kluyveromyces lactis* XP1461. Enzyme Microb Technol. 2015;77:68-77.

3. Zheng RC, Ge Z, Qiu ZK, Wang YS, Zheng YG. Asymmetric synthesis of (*R*)-1,3-butanediol from 4-hydroxy-2-butanone by a newly isolated strain *Candida krusei* ZJB-09162. Appl Microbiol Biotechnol. 2012;94(4):969-76.

4. Xue YP, Tian FF, Ruan LT, Liu ZQ, Zheng YG, Shen YC. Concurrent obtaining of aromatic (*R*)-2-hydroxyacids and aromatic 2-ketoacids by asymmetric oxidation with a newly isolated *Pseudomonas aeruginosa* ZJB1125. J Biotechnol. 2013;167(3):271-8.

5. Wang Y-J, Liu X-Q, Luo X, Liu Z-Q, Zheng Y-G. Cloning, expression and enzymatic characterization of an aldo-keto reductase from *Candida albicans* XP1463. J Mol Catal B: Enzym. 2015;122:44-50.
