# Supplementary material for: Efficient biosynthesis of ethyl (R)-4-chloro-3-hydroxybutyrate using a stereoselective carbonyl reductase from Burkholderia gladioli
Source: BMC Biotechnol. 2016 Oct 18;16:70. doi: 10.1186/s12896-016-0301-x (PMC5070160; doi:10.1186/s12896-016-0301-x)
Supplement: Additional file 2: Table S2. — Results of screening stereoselective carbonyl reductases for direct asymmetric reduction of ethyl 4-chloro-3-oxobutanoate. (DOCX 18 kb) [file 12896_2016_301_MOESM2_ESM.docx]

**Additional file 2: Table S2.** Results of screening stereoselective carbonyl reductases for direct asymmetric reduction of ethyl 4-chloro-3-oxobutanoate.

| Enzyme | GenBank accession no | Specificity activity  (U mg CFE^-1^)^a^ | CHBE |
| --- | --- | --- | --- |
| *Bg*ADH1 | YP_004360366.1 | 1.002 | >99% (*S*) |
| *Bg*ADH2 | YP_004348055.1 | 1.159 | >99% (*S*) |
| ***Bg*ADH3** | **AEA63541** | **1.137** | **>99% (*R*)** |
| *Bg*ADH4 | YP_004350148.1 | - | - |
| *Bg*ADH5 | YP_004349253.1 | 0.91 | >99% (*S*) |
| *Bg*ADH6 | YP_004349971.1 | - | - |
| *Bg*ADH7 | YP_004359365.1 | <0.1 | >99% (*S*) |
| *Bg*ADH8 | YP_004348092.1 | - | - |
| *Bg*ADH9 | YP_004350149.1 | - | - |
| *Bg*ADH10 | YP_004360275.1 | - | - |
| *Bg*ADH11 | YP_004350049.1 | - | - |
| *Bg*ADH12 | YP_004350434.1 | - | - |
| alco-deh1 | AEV88430 | - | - |
| alc-deh2 | AEV87398 | - | - |
| alc-deh3 | AEV85156 | - | - |
| alc-deh4 | WP_014689145 | - | - |
| alc-deh5 | AEV82242 | - | - |
| alc-deh5-NADP | AEV85657 | - | - |
| SDR1 | AEV85158 | - | - |
| SDR2 | AEV84597 | - | - |
| SDR3 | AEV83426 | - | - |
| Zn-alc-deh1 | WP_014689772 | - | - |
| LmADH | CDX66786.1 | - | - |
| CR125 | AAP94029.1 | 0.95 | >99% (*S*) |
| 313 | NP_010159.1 | 0.86 | >99% (*S*) |
| CDS-CR | NP_010159.1 | - | - |
| PlADH1 | YP_001413892.1 | - | - |
| *Cp*SCR | ACQ99345 | 1.48 | >99% (*S*) |
| PlADH2 | YP_001413036.1 | - | - |
| GDH1 | WP_013081865.1 | - | - |
| GDH2 | WP_003246720.1 | - | - |
| GDH4 | EEL32290.1 | - | - |
| GDH5 | WP_011198102.1 | - | - |
| GDH6 | ACB59697.1 | - | - |
| FDH1 | AF004096 | - | - |

^a^ CFE, crude frozen enzyme.
