# Supplementary material for: Efficient biosynthesis of ethyl (R)-4-chloro-3-hydroxybutyrate using a stereoselective carbonyl reductase from Burkholderia gladioli
Source: BMC Biotechnol. 2016 Oct 18;16:70. doi: 10.1186/s12896-016-0301-x (PMC5070160; doi:10.1186/s12896-016-0301-x)

**Additional file 3: Figure S1.** Amino acid sequence alignments of short-chain alcohol dehydrogenase/reductase using ESPript 3.0. Sequences are *Burkholderia gladioli* from this study (*Bg*ADH3, GenBank Accession No. AEA63541), *Gluconobacter oxydans* 621H (Gox2036, GenBank Accession No. AAW61772.1), *Lactobacillus brevis* (*Lb*ADH, GeneBank Accession No. CAD66648.1), *Salmonella enteric* (Ygha, PDB: 3R3S_A), and *Bacillus anthracis* (*Ba*ADH, PDB: 3I3O_A). The cofactor-binding motifs in the SDRs, TGXXXGXG and PG, are highlighted in orange and triangle while the residues of the catalytic tetrad (N, S, Y, and K) are highlighted in green.


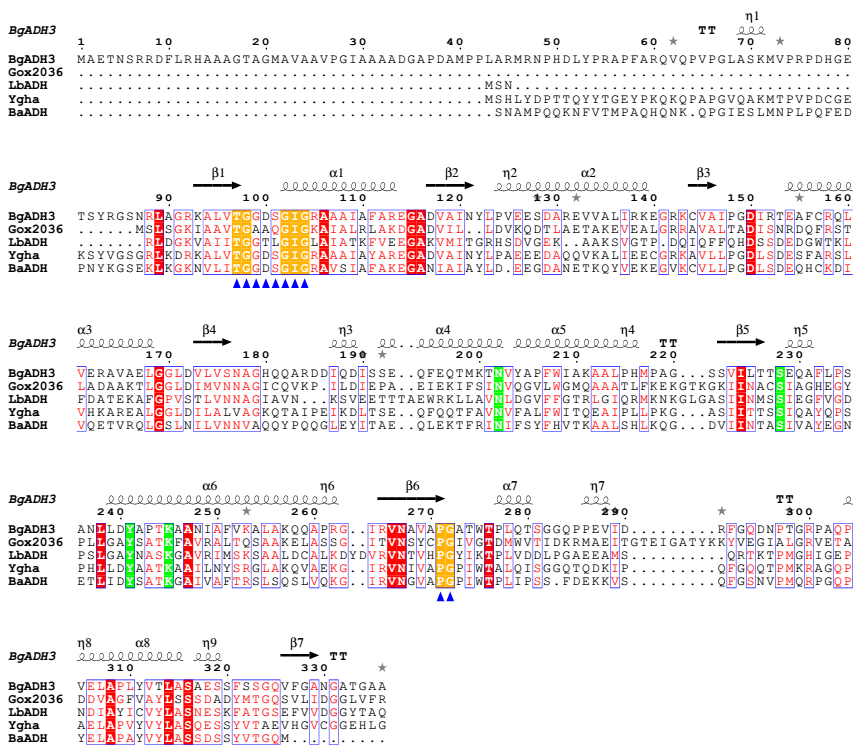

Supplement: Additional file 3: Figure S1. — Amino acid sequence alignments of short-chain alcohol dehydrogenase/reductase using ESPript 3.0. Sequences are Burkholderia gladioli from this study (BgADH3, GenBank Accession No. AEA63541), Gluconobacter oxydans 621H (Gox2036, GenBank Accession No. AAW61772.1), Lactobacillus brevis (LbADH, GeneBank Accession No. CAD66648.1), Salmonella enteric (Ygha, PDB: 3R3S_A), and Bacillus anthracis (BaADH, PDB: 3I3O_A). The cofactor-binding motifs in the SDRs, TGXXXGXG and PG, are highlighted in orange and triangle while the residues of the catalytic tetrad (N, S, Y, and K) are highlighted in green. (DOCX 212 kb) [file 12896_2016_301_MOESM3_ESM.docx]
