# Supplementary material for: Efficient biosynthesis of ethyl (R)-4-chloro-3-hydroxybutyrate using a stereoselective carbonyl reductase from Burkholderia gladioli
Source: BMC Biotechnol. 2016 Oct 18;16:70. doi: 10.1186/s12896-016-0301-x (PMC5070160; doi:10.1186/s12896-016-0301-x)
Supplement: Additional file 4: Table S3. — Effect of metal ions on the activity of recombinant BgADH3. (DOCX 17 kb) [file 12896_2016_301_MOESM4_ESM.docx]

**Additional file 4: Table S3.** Effect of metal ions on the activity of recombinant *Bg*ADH3^a^.

| Reagent | Relative activity (%) | Reagent | Relative activity (%) |
| --- | --- | --- | --- |
| Fe^2+^ | 98 ± 6.5 | Mn^2+^ | 83 ± 1.9 |
| Ni^2+^ | 95 ± 5.7 | Zn^2+^ | 102 ± 5.1 |
| Fe^3+^ | 102 ± 5.6 | Co^2+^ | 109 ± 7.3 |
| Ca^2+^ | 113 ± 6.1 | Mg^2+^ | 102 ± 6.5 |
| Ba^2+^ | 54 ± 2.9 | Ag^+^ | 25 ± 3.9 |
| Cu^2+^ | 106 ± 5.2 | EDTA-Na_2_ | 99 ± 6.5 |

^a^ The activities were determined under standard assay conditions after incubation with metal ions (2 mM) at 30 ^o^C for 30 min. The activity in the absence of metal ions was recorded as 100%. All reactions were repeated in triplicate.
