# Supplementary material for: Efficient biosynthesis of ethyl (R)-4-chloro-3-hydroxybutyrate using a stereoselective carbonyl reductase from Burkholderia gladioli
Source: BMC Biotechnol. 2016 Oct 18;16:70. doi: 10.1186/s12896-016-0301-x (PMC5070160; doi:10.1186/s12896-016-0301-x)
Supplement: Additional file 5: Table S4. — GC and HPLC analysis of the ee or de values of products. (DOCX 19 kb) [file 12896_2016_301_MOESM5_ESM.docx]

**Additional file 5: Table S4**. GC and HPLC analysis of the *ee* or *de* values of products.

| Product | Column^a^ | Conditions | Retention time (min) | | |
| --- | --- | --- | --- | --- | --- |
|  |  |  | Substrate | (*S*)-P | (*R*)-P |
| P**1** | BGB-174 | 110 ^o^C, 5 ^o^C/min, 160 ^o^C; Inc./dec. 240 ^o^C; helium | 6.6 | 7.7 | 7.9 |
| P**2** | BGB-174 | 110 ^o^C, 5 ^o^C/min, 160 ^o^C Inc./dec. 240 ^o^C; helium | 7.8 | 8.8 | 9.2 |
| P**3** | OJ-H | *n*-hexane/isopropanol (97:3, v/v); flow rate: 0.2 mL/min; 254 nm | 9.1 | 18.9 | 20.2 |
| P**4** | OJ-H | *n*-hexane/isopropanol (97:3, v/v); flow rate: 0.2 mL/min; 254 nm | 10.3 | 19.4 | 21.2 |
| P**5** | OJ-H | *n*-hexane/isopropanol (97:3, v/v); flow rate: 0.2 mL/min;254 nm | 13.3 | 25.0 | 41.7 |
| P**6** | BGB-174 | 140 ^o^C; Inc./dec. 240 ^o^C; helium | 5.1 | 6.3 | 6.8 |
| P**7** | BGB-174 | 140 ^o^C; Inc./dec. 240 ^o^C; helium | 3.5 | 5.8 | 6.1 |
| P**8** | BGB-174 | 140 ^o^C; Inc./dec. 240 ^o^C; helium | 3.6 | 6.1 | 6.3 |
| P**9** | OJ-H | *n*-hexane/isopropanol (95:5); flow rate: 0.2 mL/min; 254 nm | 9.6 | 11.4 | 12.1 |
| P**10** | BGB-174 | 110 ^o^C, 20 min, 5 ^o^C/min, 160 ^o^C; Inc./dec. 240 ^o^C; helium | 10.8 | 24.4 | 23.9 |
| P**11** | BGB-174 | 110 ^o^C, 20 min, 5 ^o^C/min, 160 ^o^C; Inc./dec. 240 ^o^C; helium | 6.2 | 19.3 | 18.9 |
| P**12** | BGB-174 | 110 ^o^C, 0.5 ^o^C/min, 125 ^o^C; Inc./dec. 240 ^o^C; helium | 7.1 | 25.0 | 24.7 |
| P**13** | BGB-174 | 110 ^o^C, 25 min, 5 ^o^C/min, 160 ^o^C, 2 min; Inc./dec. 240 ^o^C; helium | 9.3 | 34.6 | 34.4 |
| P**14** | BGB-174 | 120 ^o^C; Inc./dec. 240 ^o^C; helium | 3.8 | 7.1 | 6.8 |
| P**15** | AD-H | *n*-hexane/isopropanol (95:5); flow rate: 1.0 mL/min; 218 nm | 4.9 | 8.1 | 7.9 |
| P**16** | ODS | acetonitrile/water (1:3, v/v); flow rate: 1.0 mL/min; 220 nm | 11.4 | 10.3 | 9.8 |
| P**17** | ODS | acetonitrile/water (1:3, v/v); flow rate: 1.0 mL/min; 220 nm | 17.1 | 14.2 | 11.4 |

^a^ BGB-174 (30 m × 0.25 mm × 0.25 µm; BGB Analytik); Chiralcel OJ-H column (2.1 × 150 mm; Daicel Chemical Ind. Ltd., Japan); Chiralpak AD-H column (4.6 × 150 mm; Daicel Chemical Ind. Ltd., Japan); Hypersil ODS column (4.6 mm × 250 mm, Thermo, USA).
