# Supplementary material for: Efficient biosynthesis of ethyl (R)-4-chloro-3-hydroxybutyrate using a stereoselective carbonyl reductase from Burkholderia gladioli
Source: BMC Biotechnol. 2016 Oct 18;16:70. doi: 10.1186/s12896-016-0301-x (PMC5070160; doi:10.1186/s12896-016-0301-x)
Supplement: Additional file 6: Figure S2. — 1H NMR of the isolated (R)-CHBE (500 MHz, CDCl3). Figures S3. 13C NMR of the isolated (R)-CHBE (126 MHz, CDCl3). (DOCX 1122 kb) [file 12896_2016_301_MOESM6_ESM.docx]

**Additional file 6: Figure S2.** ^1^H NMR of the isolated (*R*)-CHBE (500 MHz, CDCl_3_).

**Additional file 6: Figure S3.** ^13^C NMR of the isolated (*R*)-CHBE (126 MHz, CDCl_3_).
