# Supplementary material for: Efficient biosynthesis of ethyl (R)-4-chloro-3-hydroxybutyrate using a stereoselective carbonyl reductase from Burkholderia gladioli
Source: BMC Biotechnol. 2016 Oct 18;16:70. doi: 10.1186/s12896-016-0301-x (PMC5070160; doi:10.1186/s12896-016-0301-x)
Supplement: Additional file 7: Table S5. — Primers from Burkholderia gladioli CCTCC M 2012379 for PCR amplification. (DOCX 18 kb) [file 12896_2016_301_MOESM7_ESM.docx]

**Additional file 7: Table S5.** Primers from *Burkholderia gladioli* CCTCC M 2012379 for PCR amplification^a^.

| Primers | Oligonucleotide sequences |
| --- | --- |
| *Bg*ADH1 | *Bg*ADH1-F: 5’-tctagaGGTCGTTCGATCAATCTGGAAGGCAAGG-3’  *Bg*ADH1-R: 5’-ctcgagTGCGAGCCCGAATCCGTCGTCG-3’ |
| *Bg*ADH2 | *Bg*ADH2-F: 5’-ccatggAGCAAGCGGCTGGAAGGCAAGG-3’  *Bg*ADH2-R: 5’-ctcgagGACCTGGGCCTGGCCGCCG-3’ |
| *Bg*ADH3 | *Bg*ADH3-F:5’-ccatggCCGAAACCAATTCGCGACGTGATTTC-3’  *Bg*ADH3-R: 5’-ctcgagGGCGGCGCCCGTCGCGCCGTTC-3’ |
| *Bg*ADH4 | *Bg*ADH4-F: 5’-ccatggATGACGACGATCGGAACCC-3’  *Bg*ADH4-R: 5’-aagcttTTACATCGCGCACAGGC-3’ |
| *Bg*ADH5 | *Bg*ADH5-F: 5’-ccatggGCAGACGTCAACAGCCTGTTC-3’  *Bg*ADH5-R: 5’-ctcgagGACCGTGCTGGTGAGGCC-3’ |
| *Bg*ADH6 | *Bg*ADH6-F: 5’-ccatggAACTGCAGGGAAAACACGCTC -3’  *Bg*ADH6-R: 5’-aagcttTCAGGCGCCGCCCCATTG -3’ |
| *Bg*ADH7 | *Bg*ADH7-F: 5’- ccatggCCCGCTTGCAAGGC -3’  *Bg*ADH7-R: 5’- aagcttTCAGCCGTTCAACATCCGC -3’ |
| *Bg*ADH8 | *Bg*ADH8-F: 5’-ccatggATTTGAACCTGCAGGACAGGATCGTG -3’  *Bg*ADH8-R: 5’-aagcttTCAGCTGATCGCGCGATCGAGGTGG -3’ |
| *Bg*ADH9 | *Bg*ADH9-F: 5’-ccatggCTGCACCCAATCTGTTCGAC-3’  *Bg*ADH9-R: 5’-aagcttCTATACCGACAGATATCCGCCGTC-3’ |
| *Bg*ADH10 | *Bg*ADH10-F: 5’-ccatggATCTGGGTTTGAACACGAAG-3’  *Bg*ADH10-R: 5’-aagcttTCAGATGCTGGCGACCAGACC-3’ |
| *Bg*ADH11 | *Bg*ADH11-F: 5’-ccatggATCTGGAGATAGCCGGC-3’  *Bg*ADH11-R: 5’-aagcttTCAGACGCTCTGGATATATCCGC-3’ |
| *Bg*ADH12 | *Bg*ADH12-F: 5’-ccatggCACAACGCAGCTTCCTCG -3’  *Bg*ADH12-R: 5’-aagcttCTAGAACGCGCGCCGGC -3’ |

^a^ *Bg*ADH1, *Bg*ADH2, and *Bg*ADH5 have been reported by ref. [[1](#_ENREF_1)].

1. Chen X, Zheng YG, Liu ZQ, Sun LH. Stereoselective determination of 2-benzamidomethyl-3-oxobutanoate and methyl-2-benzoylamide-3-hydroxybutanoate by chiral high-performance liquid chromatography in biotransformation. J Chromatogr B. 2015;974:57-64.
